# *Arabidopsis* Coexpression Tool: a tool for gene coexpression analysis in *Arabidopsis thaliana*

**DOI:** 10.1016/j.isci.2021.102848

**Published:** 2021-07-10

**Authors:** Vasileios L. Zogopoulos, Georgia Saxami, Apostolos Malatras, Antonia Angelopoulou, Chih-Hung Jen, William J. Duddy, Gerasimos Daras, Polydefkis Hatzopoulos, David R. Westhead, Ioannis Michalopoulos

**Affiliations:** 1 Centre of Systems Biology, Biomedical Research Foundation, Academy of Athens, Athens 11527, Greece; 2 Center for Research in Myology, Sorbonne Université, Paris 75013, France; 3 Department of Biotechnology, Agricultural University of Athens, Athens 11855, Greece; 4 Cold Spring Biotech Corp, Da Hu Science Park, New Taipei City, Taiwan; 5 Northern Ireland Centre for Stratified Medicine, Altnagelvin Hospital Campus, Ulster University, Londonderry BT52 1SJ, UK; 6 School of Molecular and Cellular Biology, Faculty of Biological Sciences, University of Leeds, Leeds LS2 9JT, UK

**Keywords:** Plant genetics, Bioinformatics, Plant bioinformatics

## Abstract

Gene coexpression analysis refers to the discovery of sets of genes which exhibit similar expression patterns across multiple transcriptomic data sets, such as microarray experiment data of public repositories. *Arabidopsis* Coexpression Tool (ACT), a gene coexpression analysis web tool for *Arabidopsis thaliana*, identifies genes which are correlated to a driver gene. Primary microarray data from ATH1 Affymetrix platform were processed with Single-Channel Array Normalization algorithm and combined to produce a coexpression tree which contains ∼21,000 *A. thaliana* genes. ACT was developed to present subclades of coexpressed genes, as well as to perform gene set enrichment analysis, being unique in revealing enriched transcription factors targeting coexpressed genes. ACT offers a simple and user-friendly interface producing working hypotheses which can be experimentally verified for the discovery of gene partnership, pathway membership, and transcriptional regulation. ACT analyses have been successful in identifying not only genes with coordinated ubiquitous expressions but also genes with tissue-specific expressions.

## Highlights

• 3500 primary microarray data from ATH1 Affymetrix chip were normalized using SCAN
• A tree with 21,287 *Arabidopsis thaliana* gene coexpression was constructed using UPGMA
• ACT is a web tool that identifies genes which are coexpressed to a driver gene
• ACT performs biological term enrichment analysis in coexpressed gene tree clades

## Graphical abstract

**Figure:**
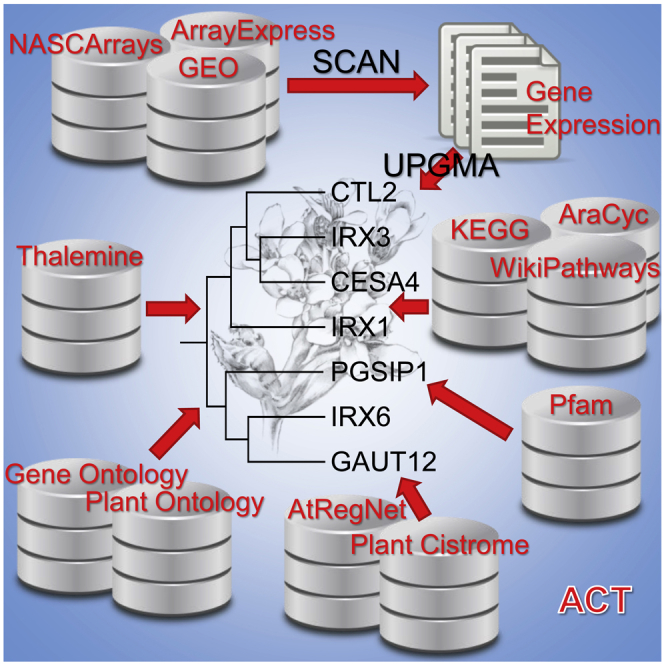

## Introduction

The introduction of microarray technology (Schena et al., 1995) enabled the study of multiple mRNA expression levels from a biological sample. Researchers are urged to share the primary and processed data of their microarray experiments, along with details of the experimental procedures, to public repositories, such as Gene Expression Omnibus (GEO) (Barrett et al., 2013) and ArrayExpress (AE) (Kolesnikov et al., 2015). The metadata of each microarray experiment are stored in repositories, in a Minimal Information About Microarray Experiments (MIAME) (Brazma et al., 2001) – compliant manner. As such, not only unnecessary repetitions of experiments are minimized but also microarray data are available. Over the past 25 years, ∼3.5 million and ∼2.5 million sample data have been stored in GEO and AE, respectively. Microarray preprocessing algorithms have been optimized and refined through the years, as has genome and transcriptome knowledge advanced, enabling primary data reuse and reanalysis that increase result reliability.

There are two main ways to assign biological functions to genes using microarrays: The first one is the differential expression analysis where gene expression levels from samples of two or more biological conditions are compared to identify genes with statistically significant differences in expression levels. The second approach includes analyses with combined microarray experiments such as meta-analysis and coexpression. Coexpression is usually larger in scale and involves the study of gene expression in a multitude of samples from the same organism (Michalopoulos et al., 2012). Genes with similar expression patterns tend to participate in related biological processes (Petereit et al., 2016). The most efficient way to study global gene coexpression is based on the transcriptomic data analysis from a subset of samples which contain the best representatives of each tissue or cell type, referred to as “condition-independent” coexpression analysis (Usadel et al., 2009). Due to the recent accumulation of large amounts of transcriptomic data, a series of gene coexpression networks (GCNs) have been developed (Serin et al., 2016). GCNs allow the study of the coexpression patterns of multiple genes in different biological conditions. Coexpression networks depict the degree of similarity between the expression profiles of all genes, in a particular set of biological samples which may derive from different tissues, developmental stages, or environmental conditions. As genes under common regulatory control are likely functionally linked, the construction of gene coexpression networks contributes to the identification of functional interactions between genes, as well as the assignment of new roles to genes (He and Maslov, 2016).

*Arabidopsis thaliana* is a model plant organism that has largely contributed to molecular biology and developmental genetics of plants, among others (Woodward and Bartel, 2018). *A. thaliana* possesses one of the smallest genomes among plants, about 130 MBp and 5 diploid chromosomes (Arabidopsis Genome, 2000). The latest version of Araport (Cheng et al., 2017) estimates the number of genes (including transposable elements and pseudogenes) to ∼33,000, providing a solid basis of gene information for additional research. Furthermore, the plant's short life cycle, small size, and ease in conditional cultivation and genetic manipulation make it a perfect candidate for microarray experiments. As a result, the wealth of microarray data allowed multiple secondary analyses. There are several gene coexpression databases and public tools for *A. thaliana* including ACT (Jen et al., 2006; Manfield et al., 2006), Atted-II (Obayashi et al., 2018), AraNet v2 (Lee et al., 2015), EXPath 2.0 (Chien et al., 2015), PLANEX (Yim et al., 2013), Genevestigator (Hruz et al., 2008), SeedNet (Bassel et al., 2011), FlowerNet (Pearce et al., 2015), AtGGM2014 (Ma et al., 2015), and GEM2Net (Zaag et al., 2015), the latter four employing coexpression networks in their approach. We introduce a new version of the ACT website, originally developed over 15 years ago, bringing the tool up to date with the latest discoveries in microarray analysis and *A. thaliana* gene-related data.

### Design

The development of a new version of the ACT tool was prompted by the need to perform a major upgrade on the original defunct tool (Table 1). The original ACT version (Jen et al., 2006; Manfield et al., 2006) was based on 322 (out of ∼1400) randomly selected microarray samples from NASCArrays (Craigon et al., 2004), normalized with MAS5.0 algorithm (Hubbell et al., 2002) along with default Affymetrix chip description file (CDF) mapping 22,746 probe sets to more than 22,000 genes. The new version of ACT web tool is based on 3500 microarray samples, automatically selected as representatives of 19,887 samples which were rigorously quality controlled, normalized with the novel Single-Channel Array Normalization (SCAN) algorithm (Piccolo et al., 2012) in accordance with the latest BrainArray CDF (Dai et al., 2005), producing expression values for 21,287 probes sets, each of which corresponds to a unique gene.

**Table 1:** Comparison of the old and new versions of ACT

Category	Original ACT	New ACT
Available samples	~1400	19,887
Selected samples	322	3500
Sources	NASCArrays	NASCArrays, GEO, ArrayExpress
Quality control	No	Yes
Normalisation algorithm	MAS5.0	SCAN
Chip description file	Default Affymetrix CDF	Latest Brainarray CDF
Output	Gene Coexpression List, Gene Cliques, Co-correlation scatterplot	Gene Coexpression Cladogram
Enrichment analysis	Words, Gene Ontology	Gene Ontology, Plant Ontology, Biological Pathways, Protein Families, experimentally verified Transcription Factors

Correlation between all probe set pairs was performed by calculating their Pearson correlation coefficients (r-values) (Pearson, 1895), in both versions. Old ACT was producing a gene list with the most correlated genes to a gene of interest, sorted in descending order of the precalculated pairwise r-values between the query gene and the rest of the genes. The coexpressed genes were containing clickable links, allowing each gene to become the driver gene for a new analysis. Judging from the already visited links in the coexpression gene list results, users observed that the top coexpressed genes were also tending to be coexpressed amongst themselves. Nevertheless, it was difficult to keep track of the top coexpression partners, after iteratively navigating the tool. To this end, graph-theory-based “Clique Finder” functionality was implemented: Genes were being treated as vertices and their pairwise correlations as edges. The top 100 coexpressed genes with a driver gene were being used to create a complete graph using all possible pairwise r-values. The edges were being pruned to keep only top 50%. Bron–Kerbosch algorithm (Bron and Kerbosch, 1973) was discovering the possible maximal cliques (subgroups of genes which are all connected to each other) of that gene network. Finally, overlapping cliques were being clustered to form subnetworks of closely associated genes. Old ACT could also detect the most correlated genes to 2 functionally related genes of interest through a scatter “cocorrelation” plot that was depicting the pairwise r-values between the 2 user-defined genes and each of the other genes. Genes having higher r-values between themselves and the 2 genes of interest, than the R value between those 2 genes, were being considered coexpressed to the gene pair. To overcome the limitations of the original ACT such as user interface complexity, dependency on arbitrary cutoff values (coexpression lists and Clique Finder) or flawed biological assumptions (cocorrelation plot), UPGMA hierarchical clustering method (Sokal and Michener, 1958) was used in the new version. Hierarchical clustering takes into consideration all Pearson correlation coefficients of each gene pair, transformed to distances. Thus, it constitutes an objective way to group coexpressed genes. ACT depicts the *Arabidopsis* global coexpression landscape by using an interactive cladogram, which contains the driver gene and its coexpressed genes in neighboring leaves. ACT gives the users the choice to find the optimal coexpression gene list through increasing and decreasing the tree size, by observing the changes in the tree topology and the biological enrichment p values, which provide hints of the preferable tree size. The website was implemented using modern technologies, offering a user-friendly design, minimizing unnecessary user interactions.

In the old ACT version, users could perform word or Gene Ontology enrichment analysis on the produced gene lists. In the new version, the variety and quality of available enrichment analyses has significantly increased. Enrichment categories include gene ontologies from Gene Ontology (Gene Ontology Consortium, 2021), plant ontologies from Planteome (Cooper et al., 2018), biological pathways from KEGG Pathways (Kanehisa and Goto, 2000), AraCyc (Schlapfer et al., 2017) and WikiPathways (Martens et al., 2021), experimentally confirmed transcription factor gene targets from AtRegNet (Yilmaz et al., 2011) and Plant Cistrome Database (O'Malley et al., 2016) and protein domains from Pfam (Mistry et al., 2021).

## Results

### Ribosomal proteins

The ribosomal subunit in *A. thaliana* consists of 80 ribosomal proteins (r-proteins). A total of 249 ribosomal protein genes are classified into 80 different r-protein types. None of these genes are single copy ones, meaning that most of the r-proteins are encoded by three or four expressed genes (Barakat et al., 2001). *AT4G13170*, a gene coding for an L13 ribosomal protein, was selected as the driver gene for an ACT analysis. The default 5 ancestral nodes coexpression subtree had a total of 134 gene leaves (Figure S1). The tree was also viewed by iTOL (Letunic and Bork, 2019) (Figure S2). Most correlated genes are structural constituents of ribosome (Table S1). To verify that this finding is statistically significant, biological term enrichment analyses were performed (Table 2). The enriched terms of all three aspects of Gene Ontology are indeed related to ribosome and translation process, with very low false-discovery-rate (FDR)-adjusted p values, ranging from ∼10^−176^ to ∼10^−156^. KEGG pathway analysis similarly suggested a ribosomal role and Pfam analysis showed an enrichment of ribosomal protein families. Terms related to “cotyledon” and “embryo structure” emerged as overrepresented plant anatomy terms in plant ontology, while a term combining the last two terms (“plant embryo cotyledonary stage”) appeared as overrepresented in plant structure developmental stage analysis. Transcription factor enrichment analysis using both AtRegNet and Plant Cistrome Database revealed two transcription factors, *AT1G72740* (*TRB5*) and *TRB2*, which belong to the homeodomain-like/winged-helix DNA-binding family of proteins. This finding is in accordance with the discovery that TRB family transcription factors regulate genes involved in the assembly of the translation mechanism in plants (Schrumpfova et al., 2016).

**Table 2:** All the important enrichment results for *AT4G13170*

Enrichment summary for *AT4G13170*			
Category	p value	Term ID	Description
GO Biological process	1.2⋅10^−176^	GO: 0006414	translational elongation
	7.0⋅10^−156^	GO: 0006412	Translation
GO Molecular function	5.9⋅10^−194^	GO: 0003735	structural constituent of ribosome
GO Cellular component	2.8⋅10^−221^	GO: 0022626	cytosolic ribosome
PO Plant anatomy	1.6⋅10^−25^	PO: 0020030	Cotyledon
	1.6⋅10^−25^	PO: 0025099	embryo plant structure
PO Plant structure development stage	8.4⋅10^−17^	PO: 0001078	plant embryo cotyledonary stage
KEGG	8.6⋅10^−155^	KEGG: ath03010	Ribosome - Arabidopsis thaliana (thale cress)
AtRegNet	1.0⋅10^−22^	AT1G72740	Homeodomain-like/winged-helix DNA-binding family protein
	7.5⋅10^−13^	TRB2	Homeodomain-like/winged-helix DNA-binding family protein
Pfam	1.0⋅10^−6^	Pfam: PF01248	Ribosomal_L7Ae

Most of the terms describe ribosome properties. See also Figures S1 and S2 and Table S1.

The gene list of the subtree was used as input in a WebGestalt (Liao et al., 2019) GO biological process overrepresentation analysis, using the list of the 21,273 genes which are studied in ATH1 microarray chip, as reference. The results coincided with ACT's enrichment analysis, showing “translation” as statically significant overrepresented term. A BioGrid (Oughtred et al., 2021) Protein-Protein Interaction Network Topology-based Analysis was also conducted, using the network expansion method with default parameters. The resulting network (Figure S3) revealed polyubiquitin 3 (*UBQ3*) as one of the top-ranking neighbors, while it should be noted that ubiquitin extension protein 1 (*UBQ1*) is one of the coexpressed genes in the subtree.

A text mining-based protein–protein association network was created in STRING (Szklarczyk et al., 2021), using the same gene list (Figure S4). Although three genes were not recognized, the resulting 131 gene network displays high connectivity amongst the nodes (network density (Coleman and Moré, 1983) 0.37).

A ThaleMine (Krishnakumar et al., 2015) list analysis of the 134 coexpressed genes was performed, which revealed the same enriched GO terms albeit with lower p values (∼10^−109^ for “translation”) compared with ACT. An additional analysis of interest is the Publication Enrichment, with the top two publications (Barakat et al., 2001; Carroll et al., 2008) exhibiting p value between 10^−256^ and 10^−235^. Both publications are related to the cytoplasmic ribosomal proteins.

An aGoTool functional enrichment analysis, using Flame (Thanati et al., 2021), revealed that this ACT gene list contained 114 UniProt (UniProt Consortium, 2021) ribosomal proteins (p value: 6.12⋅10^−4^) and 25 InterPro (Blum et al., 2021) ribosomal domains from 3 to 8 members each (p value range: ∼10^−2^ - ∼10^−3^).

### Heat shock proteins

Heat shock proteins (HSP) are a family of proteins expressed in response to stressful conditions. Heat shock protein 101 (*HSP101*) gene, belonging in the HSP100 family which is responsible for high temperature survival in *A. thaliana* (Tonsor et al., 2008), was used as input in ACT. After expanding the initial resulting subtree to 11 ancestral nodes with a total of 44 gene leaves, the GO biological process analysis pointed the “resistance to heat” as top-ranking term and the Pfam analysis sorted more than half of the genes in coding proteins of the *HSP20* family (Table 3). In addition, AtRegNet analysis discovered 5 transcription factors targeting the genes of the subtree: The top-ranked transcription factor, *AT3G09735*, is poorly annotated, while the consequent transcription factors, *HSF3*, *HSFB2A*, *HSFC1,* and *AT-HSFB2B*, are all heat shock ones. Secondly, heat shock protein 90 (*HSP90, AT5G56030*) was selected as a driver gene. Expanding the initial tree to 66 gene leaves, GO biological process analysis showed “resistance to heat,” “response to temperature,” “response to high light intensity,” “response to abiotic stimulus,” and “protein folding” (Table S2) in accordance with *HSP90* generic protein functions (Milioni and Hatzopoulos, 1997). KEGG enrichment analysis demonstrated the term entitled “Protein processing in ER” which firmly confirms *HSP90* role as a chaperone assisting other proteins to fold properly and stabilize.

**Table 3:** *HSP101* results of the over-representation analysis

Enrichment summary for *HSP101*				
Category	p value	Term ID	Description	
GO Biological process	9.1⋅10^−45^	GO: 0009409	response to heat	
Pfam	1.5⋅10^−29^	Pfam: PF00011	Hsp20/alpha crystallin family	
	1.5⋅10^−5^	Pfam: PF00012	Hsp70 protein	
AtRegNet	2.3⋅10^−24^	AT3G09735	S1FA-like DNA-binding protein	
	5.6⋅10^−24^	HSF3	heat shock factor 3	
	2.3⋅10^−19^	HSFB2A	heat shock transcription factor B2A	
	2.9⋅10^−17^	HSFC1	heat shock transcription factor C1	
	4.9⋅10^−5^	AT-HSFB2B	winged-helix DNA-binding transcription factor family protein	

See also Table S2.

### Response to cold

**Figure 1:**
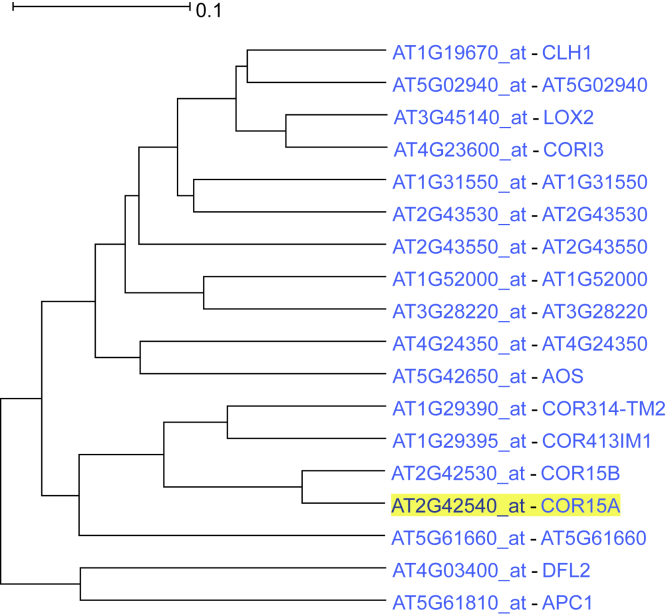
ACT output of the coexpression subtree of *COR15A* with the default 5 ancestral nodes See also Figure S3.

In *A. thaliana,* cold-regulated 15a (*COR15A*) gene enhances resistance to freezing (Artus et al., 1996; Wang and Hua, 2009). We selected *COR15A* as driver gene (Table S3). *COR15A* and its homolog, *COR15B*, were located next to each other in the resulting 18-gene-leaves coexpression subtree, along with 2 other cold-regulated genes, *COR314-TM2* and *COR413IM1* belonging to the same subclade (Figure 1). Biological process analysis showed an overrepresented “cold acclimation” attribute.

### Cell wall biogenesis

**Figure 2:**
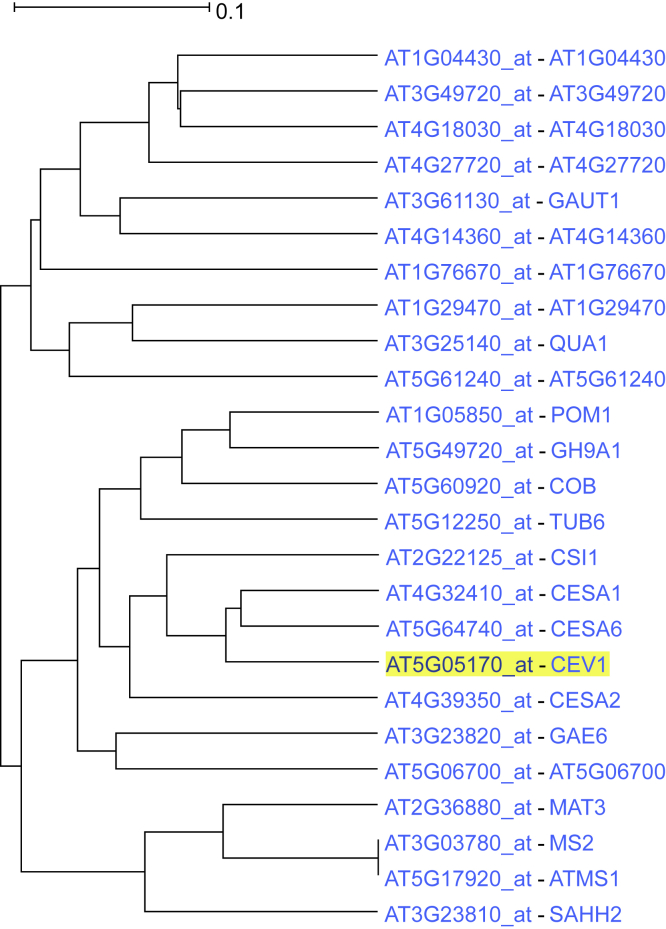
*CEV1* coexpression subtree as output by ACT expanded to 7 ancestral nodes See also Figure S2.

A member of cellulose synthase gene family, *CEV1,* was used for ACT analysis. *CEV1* is a catalytic subunit of cellulose synthase complexes involved in the primary cell wall formation (Burn et al., 2002; Daras et al., 2009). The subtree was expanded to 7 nodes (Figure 2) and showed coexpression with other cellulose synthase genes and proteins involved in cell expansion, such as *COB, POM1,* and cellulose synthase-interacting protein *CSI1*. Gene Ontology enrichment analysis of the coexpressed gene network for biological process demonstrated the terms “plant-type primary cell wall biogenesis,” “polysaccharide biosynthetic process,” “cellulose biosynthetic process,” and “beta-glucan biosynthetic process” as top hits (Table S4). Additionally, regarding molecular function, top hits were the terms “cellulose synthase (UDP-forming) activity,” “cellulose synthase activity,” “S-methyltransferase activity,” and “UDP-glycosyltransferase activity” corroborating the role of the genes in this network. Gene Ontology analysis in terms of cellular component showed overrepresentation for “*trans*-Golgi network,” “Golgi subcompartment,” and “plasma membrane” supporting the function of these genes in those subcellular compartments (Wightman and Turner, 2010).

**Figure 3:**
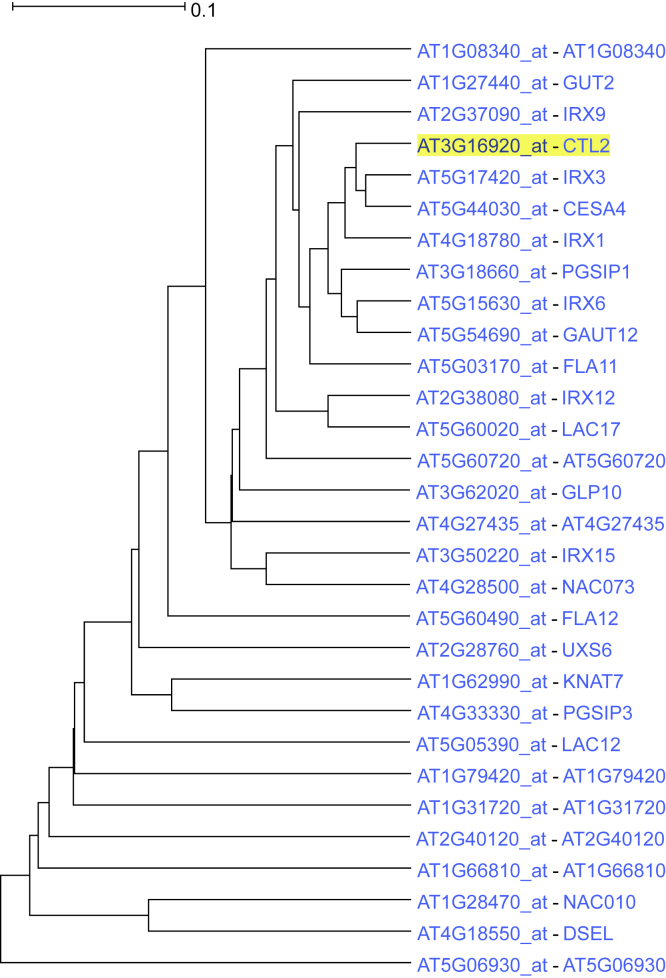
*CTL2* coexpression subtree as output by ACT The majority of the genes are related to plant-type secondary wall biogenesis.

**Figure 4:**
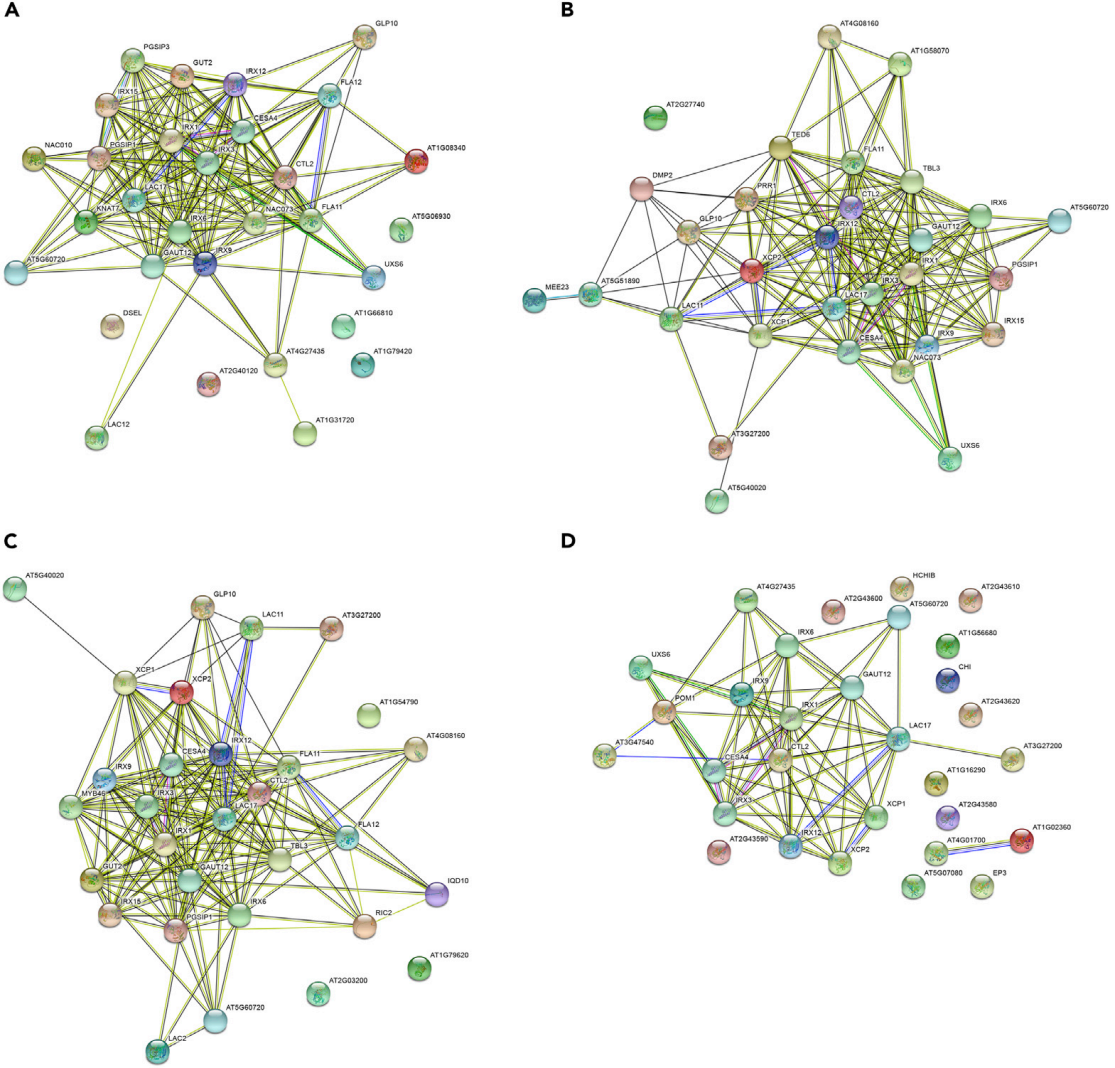
Protein-Protein Interaction networks of the *CTL2* coexpressed gene list results from different tools STRING protein networks using the coexpressed genes to *CTL2* according to ACT (A), ATTED-II (B), Genevestigator (C) and Genemania (D).

Further, chitinase-like protein 2 (*CTL2*), a gene with probable role in secondary cell wall synthesis in *A. thaliana* (Hossain et al., 2010; Koizumi et al., 2009), was used for an ACT analysis with the subtree expanded to 22 nodes (Figure 3). GO biological process enrichment analysis of the 30 coexpressed genes ranked “plant-type secondary wall biogenesis” as the top term (p value: 1.0⋅10^−30^) with “plant-type cell wall biogenesis” a close second (p value: 4.8⋅10^−25^) and AraCyc analysis also proposed cellulose biosynthesis as an enriched term (p value: 2.9⋅10^−5^). “Lignin catabolic process,” another enriched GO biological process (p value 1.5⋅10^−5^), is in accordance with the finding that a mutation of *CTL2* increases lignin accumulation in dark-grown *Arabidopsis* seedlings (Hossain et al., 2010). The cotton ortholog of *CTL2* is expressed preferentially in cells with secondary walls (Zhang et al., 2004). A protein association network of the resulting coexpression subtree leaves was created using STRING (Figure 4A). This network showed a strong connection between the driver gene and several genes in adjacent leaves of the subtree, especially those of the IRX family of proteins. Finally, AtRegNet analysis showed *VND7* as the top-ranked transcription factor among other overrepresented ones. *VND7* regulates patterns of secondary cell wall deposition in vascular vessels (Yamaguchi et al., 2011) and is also known to bind to the promoters of many secondary cell wall biosynthesis genes (Taylor-Teeples et al., 2015).

We decided to use *CTL2* as gene input to ATTED-II, Genevestigator and Genemania (Franz et al., 2018). We selected the top 29 coexpressed genes from the resulting coexpression gene list of each tool, additionally including the driver gene *CTL2* for a total of 30 genes per list. We used g:Profiler (Raudvere et al., 2019) as a common enrichment analysis tool and used each coexpression gene list from the four different tools as input. The top g:Profiler Biological Process enrichment term for ACT gene list was “plant-type secondary wall biogenesis” (p value: 2.6⋅10^−30^). g:Profiler also proposed “plant-type secondary wall biogenesis” as top enriched term for ATTED-II and Genevestigator (p values: 7.6⋅10^−18^ and 8.0⋅10^−12^ respectively). The same term appeared in the Genemania g:Profiler enrichment results, albeit with a much higher p value (1.5⋅10^−5^). In addition, the individual lists were inserted into a String protein–protein interaction (PPI) network creation analysis (Figure 4) and network density (Coleman and Moré, 1983) was calculated for each graph: 0.36 for ACT, 0.42 for ATTED-II, 0.40 for Genevestigator, and 0.17 for Genemania.

### Photosynthesis

PSB28 protein is a component of photosystem II (*PSB28*), aiding in the repair and *de novo* synthesis of PSII complex proteins as a response to extreme high light-induced stress (Parrine et al., 2018). The default ACT search produced a subtree that proposed “photosynthesis” as the top biological process. We expanded the coexpression subtree to the point where minimum p values were achieved. A total of 41 ancestral nodes resulted to a 729-gene-leaves tree upon which biological term analyses were performed (Table 4). Top terms for biological process, KEGG and AraCyc analysis all showed “photosynthesis” as overrepresented, while cellular component proposed “plastid” as the plant organelle coinciding with molecular function analysis top term of “pigment binding” and plant anatomy's “cotyledon primordium.” In addition, Pfam showed chlorophyll-binding protein as a top protein family and AtRegNet discovered phytochrome interacting factor 4 (*PIF4*) as a top transcription factor.

**Table 4:** Enrichment summary table for *PSB28*

Enrichment summary for *PSB28*			
Category	p value	Term ID	Description
GO Biological process	5.9⋅10^−123^	GO: 0015979	Photosynthesis
	2.2⋅10^−74^	GO: 0019684	photosynthesis, light reaction
GO Molecular function	3.3⋅10^−12^	GO: 0031409	pigment binding
GO Cellular component	0	GO: 0044434	chloroplast part
	0	GO: 0044435	plastid part
PO Plant anatomy	2.6⋅10^−125^	PO: 0000015	cotyledon primordium
	2.6⋅10^−125^	PO: 0025432	cotyledon anlagen
KEGG	1.4⋅10^−40^	KEGG: ath00195	Photosynthesis - Arabidopsis thaliana (thale cress)
AraCyc	8.1⋅10^−24^	AraCyc: PWY-101	photosynthesis light reactions
AtRegNet	5.8⋅10^−5^	PIF4	phytochrome interacting factor 4
Pfam	1.2⋅10^−13^	Pfam: PF00504	Chlorophyll A-B binding protein

All of the over-represented terms are related to photosynthesis.

### Circadian rhythm

*LATE ELONGATED HYPOCOTYL* (*LHY*) gene plays a role in the *A. thaliana* circadian clock (Lu et al., 2009). Using *LHY* for an ACT analysis and after expanding the subtree to 8 nodes (Table 5), the top biological process enriched term in the subtree of 21 gene leaves was “rhythmic process.” KEGG pathway also proposed circadian rhythm in *Arabidopsis* as enriched term and Pfam categorized 8 of the genes as coding transcription factors that belong to the B-box zinc finger (zf-B_box) and Myb-like DNA-binding domain (Myb_DNA-binding) families. AtRegNet found timing of cab expression 1 (*TOC1*), a key clock component that integrates the environmental information to coordinate circadian responses (Perales and Mas, 2007), as a targeting transcription factor. Interestingly, two transcription factor genes, *RVE8* and *CCA1*, which bind to the promoter of *TOC1* (Farinas and Mas, 2011), were among the correlated genes.

**Table 5:** Enrichment analysis results for the *LHY* coexpression subtree after it was expanded to 7 ancestral nodes

Enrichment summary for *LHY*			
Category	p value	Term ID	Description
GO Biological process	6.8⋅10^−12^	GO: 0048511	rhythmic process
KEGG	5.6⋅10^−7^	KEGG: ath04712	Circadian rhythm - plant - Arabidopsis thaliana (thale cress)
Pfam	1.8⋅10^−11^	Pfam: PF00643	B-box zinc finger
	1.0⋅10^−4^	Pfam: PF00249	Myb-like DNA-binding domain

### Chloroplast and mitochondrial proteins

**Figure 5:**
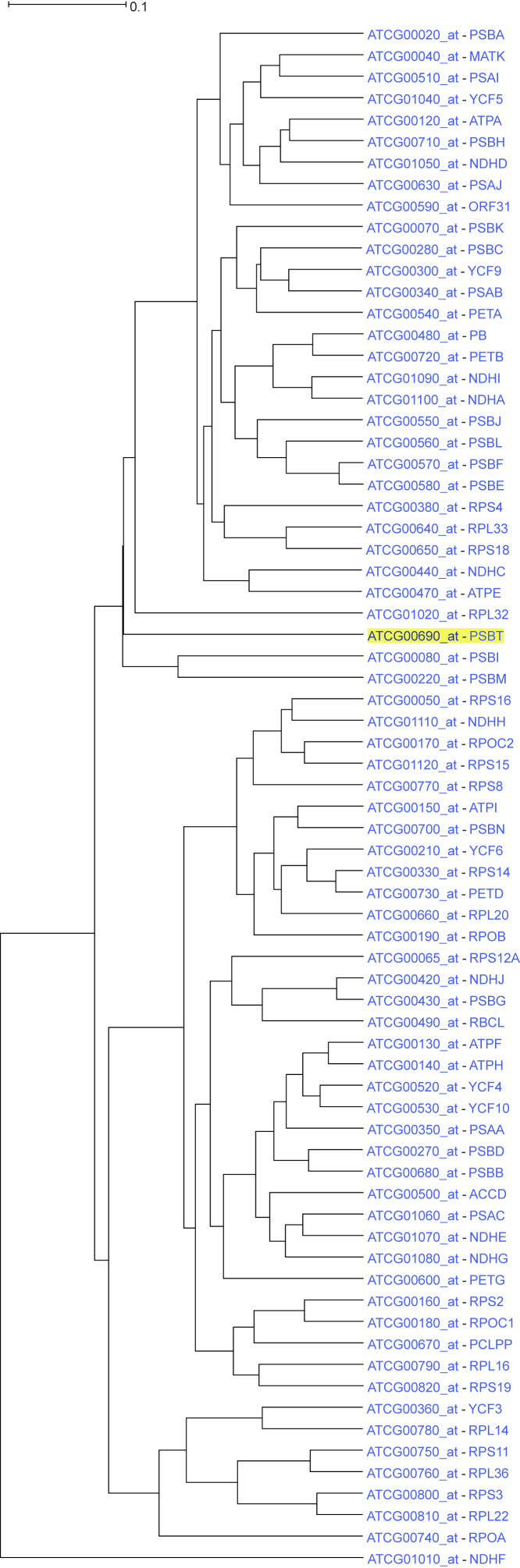
Coexpression tree containing exclusively all 72 chloroplast genes

The ATH1 genome array contains probesets for 72 chloroplast genes. Using one of those genes, photosystem II reaction center protein T, as a driver gene in ACT and reducing the ancestral nodes to 4, a coexpression tree that contained exclusively all 72 available chloroplast genes (recognized by the “ATCG” prefix of the probeset ID), was produced (Figure 5). The first seven genes were related to translation, while the rest were predominantly related to photosynthesis.

**Figure 6:**
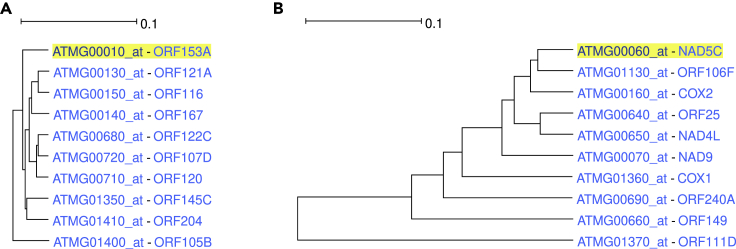
Mitochondrial genes coexpression trees (A) Coexpression tree containing 10 correlated Mitochondrial genes. All proteins are hypothetical. (B) Coexpression tree containing another 10 correlated Mitochondrial genes

The ATH1 genome array studies the expression of 27 mitochondrial genes (recognized by the “ATMG” prefix of the probeset ID). Unlike chloroplast genes, the mitochondrial ones are not located in a single subtree but are rather found in clusters. There are two subtrees containing grouped mitochondrial genes, the first with 10 and the second with 11 leaves. The first tree has only hypothetical proteins (Figure 6A) while the second one is better annotated (Figure 6B) and possesses biological process enrichments of “cellular respiration” (p value: 1.3⋅10^−7^) and “energy derivation by oxidation of organic compounds” (p value: 1.5⋅10^−7^). The rest of the mitochondrial genes are clustered in groups of two or three.

### Anther and pollen

*ABORTED MICROSPORES* (*AMS*) gene plays a role in tapetal cell development (Xu et al., 2010). It was used as a driver gene to an ACT analysis and the resulting subtree was expanded to 12 ancestral nodes (101 genes). Biological process enrichment analysis produced “pollen wall assembly” as the top-ranking term, cellular component analysis discovered “pollen coat” as an enriched term, and the top three ranking terms for plant anatomy analysis were “sporangium wall,” “tapetum,” and “anther” (Table 6).

**Table 6:** *AMS* coexpression tree major enrichment results

Enrichment summary for *AMS*			
Category	p value	Term ID	Description
GO Biological process	7.9⋅10^−22^	GO: 0010208	pollen wall assembly
GO Cellular component	1.5⋅10^−6^	GO: 0070505	pollen coat
PO Plant anatomy	9.9⋅10^−17^	PO: 0025306	sporangium wall
	1.2⋅10^−15^	PO: 0025313	Tapetum
	6.3⋅10^−15^	PO: 0009066	Anther

### Embryo development

*Embryo defective 1692 (emb1692 or Lefkothea)* gene is a nuclear-encoded RNA-binding protein, participating in chloroplast group II intron and nuclear pre-mRNA splicing. *emb1692* protein controls embryonic and postembryonic development and is mainly expressed in meristems localized to both nuclei and chloroplasts (Daras et al., 2019). ACT analysis of *emb1692* gene resulted in an expanded network of 68 nodes (Figure S5). Most of the genes belong to pentatricopeptide repeat (PPR) which is mainly involved in RNA metabolism in organelles having essential roles in their biogenesis and embryo development (Lurin et al., 2004). Since *emb1692* gene participates in chloroplast group II intron splicing, a network with PPR genes justifies the role of coexpressed genes in RNA metabolism. Gene Ontology analysis of the network for biological process demonstrated the terms “RNA modification,” “embryo development,” “seed development,” “RNA processing,” and “chloroplast RNA modification” firmly related to genes functions. In addition, enrichment summary of plant structure development stage resulted in terms related with different embryonic stages (Table 7) supporting the role of *emb1692* and its coexpressed genes to control embryonic development. Enrichment summary of PFAM showed PPR and DYW gene family as top hits. DYW family is a subgroup of PPR gene family and is essential mainly for RNA editing in organelles (Okuda et al., 2009).

**Table 7:** Enrichment summary table for *emb1692* after it was expanded to 8 ancestral nodes

Enrichment summary for *emb1692*			
Category	p value	Term ID	Description
GO Biological process	1.5⋅10^−7^	GO: 0009451	RNA modification
	1.1⋅10^−4^	GO: 0009790	embryo development
	1.1⋅10^−4^	GO: 0048316	seed development
	1.5⋅10^−4^	GO: 0006396	RNA processing
	2.2⋅10^−4^	GO: 1900865	chloroplast RNA modification
PO Plant structure developmental stage	5.9⋅10^−6^	PO: 0001078	plant embryo cotyledonary stage
	6.6⋅10^−6^	PO: 0001081	mature plant embryo stage
Pfam	1.5⋅10^−11^	Pfam: PPR_2	PPR repeat family
	1.5⋅10^−11^	Pfam: PPR	PPR repeat
	1.3⋅10^−8^	Pfam: DYW_deaminase	DYW family of nucleic acid deaminases

See also Figure S5.

We decided to use *emb1692* to compare ACT's internal enrichment analysis tool to g:Profiler. *emb1692* coexpression gene list produced by ACT was used as input for a g:Profiler enrichment analysis. Both tools proposed “RNA modification” as the top biological process term and “plastid” and “chloroplast” as top cellular component terms. The list includes 6 genes named as embryo defective: *EMB1006*, *emb1703*, *emb1688*, *EMB3120*, *EMB2729,* and *emb1692*. Thus, “embryo development” emerged as a statistically significant enriched biological process term in ACT (p value: 1.1⋅10^−4^), as it discovered 10 genes described by this term. Nevertheless, g:Profiler failed to characterize this term as significant (p value: 1.0), as it only discovered 2 genes described by this particular term.

## Discussion

New approaches that distinguish ACT from other coexpression tools for *A. thaliana*, as well as its previous version, were employed. The samples used in new version of ACT are more than 10 times the previous amount, from representative distinct tissues, selected from an even larger sample pool. As a result, the produced r-values are improved and do not contain any kind of tissue bias. While ACT similarly exclusively relies on data from a single microarray platform, tools, such as ATTED-II and EXPath, use both microarray and RNA-seq data, showing significant discrepancy between coexpression calculation based on the two data sets. The previous version of our tool used MAS5.0 single-array normalization algorithm (Hubbell et al., 2002) for data processing and normalization. Soon after the development of original ACT, though, Affymetrix suggested that MAS5.0 should be primarily used to obtain a quick report regarding the performance of the arrays and to identify any obvious problems, rather than as a main normalization method (Affymetrix, 2018; Dziuda, 2010). Instead, they suggested the submission of the final set of arrays to RMA (Irizarry et al., 2003) or PLIER (Hubbell, 2005) multi-array normalization algorithms. Most coexpression analysis tools indeed employ RMA which assumes that probe intensity value distribution across all samples is common. This assumption makes multiarray normalization algorithms unsuitable for coexpression analysis, as samples derive from different tissues or research groups. This may explain why multiarray normalization algorithms introduce a large number of correlation artifacts (false correlated gene pairs among the top most correlated ones) and single-array MAS5.0-normalized data provide by far the best platform for inferring PPIs (Lim et al., 2007). Consequently, SCAN (Piccolo et al., 2012) was used over the other single-array alternative, MAS5.0, and RMA. SCAN algorithm offers a novel normalization method that preprocesses each sample independently from each other and it performs a GC content bias correction, increasing the total signal-to-noise ratio. The use of SCAN along with the large and diverse microarray sample pool guarantees the avoidance of spurious correlations between genes, a pitfall which arises when a combination of a small number of samples and a quantile normalization algorithm is used (Usadel et al., 2009). Default Affymetrix CDF that was used to map ATH1 probe sets to genes both in original ACT and in most other coexpression tools, contains 22,746 noncontrol probe sets defined in 2002. However, 5.47% of those probe sets do not correspond to any gene and 3.82% correspond to multiple genes. Furthermore, total number of genes mapped by the array using the default CDF is 22,168, out of which the 118 are obsolete. To maintain a one-to-one relation between probes and genes, ATTED-II selected a single probe set of the outdated default Affymetrix CDF for each gene, Planex performed its own mapping programmatically, EXPath discarded all the ambiguous mappings, and AtGGM2014 used the mappings provided by TAIR (Lamesch et al., 2012). Instead, new version of ACT uses up-to-date BrainArray CDF (Dai et al., 2005) which lacks the drawbacks of the default CDF, as it ensures that each probe set corresponds to a single gene and *vice versa*, totaling 21,287 nonobsolete genes. Furthermore, BrainArray is annually updated, defining its probe sets according to the current genomic and transcriptomic knowledge.

ACT's strength lies in simplicity and focus, specifically catering for molecular biologists, producing easy-to-understand biologically relevant outputs, avoiding user information overload which characterized the original version and other coexpression tools. Furthermore, the overrepresentation of adjusted p values are presented in commonly understood numeric format (e.g. 1.0∙10^−15^) instead of the scientific numeric format (e.g. 1.0E-15), while results with p values>0.05 are omitted to prevent the inclusion of nonstatistically significant terms. The enrichment summary tables are easy to produce inside ACT, provide various descriptions for each term with links redirecting to relevant external databases and can be easily copied through any web browser.

Most tools, including ACT, perform Gene Ontology enrichment analysis. ACT enrichment analysis takes account of Gene Ontology terms of all evidence codes which describe the selected genes. On the other hand, tools such as g:Profiler omit GO terms of specific evidence codes, thus missing overrepresented terms in their analysis. Therefore, ACT reveals biologically relevant enriched GO terms which g:Profiler fails to discover, as it happened with highly informative term “embryo development,” in the case of *emb1692* coexpression list enrichment analysis. Some tools offer additional enrichment options, such as pathway analysis (EXPath, ATTED-II) or transcription element identification (ATTED-II). However, while ATTED-II identifies *bona fide cis*-elements in correlated genes without identifying the transcription factors that target those motifs, ACT identifies enriched experimentally verified transcription factors targeting coexpressed genes, an information crucial to molecular biologists as it reveals the transcription factors that orchestrate gene coregulation. This specific kind of enrichment analysis is unique to ACT which uses data of confirmed transcription factors directly targeting *A. thaliana* genes from both AtRegNet and DAP-seq experiment-based Plant Cistrome database. Moreover, the gene list of each ACT sub tree is provided in an easy-to-use format for downstream analysis. Links to STRING for creating PPI networks and ThaleMine for gene list analysis which includes bibliography enrichment, are provided through ACT.

SeedNet and GEM2Net differ in their approach, as they categorize genes through their gene expression, both positively and negatively, based on seed germination and biotic/abiotic stress, respectively. Although SeedNet provides a comprehensible coexpression network visualization while including both correlated and anticorrelated genes, it offers no enrichment analysis. Furthermore, its AGI Code-Gene Symbol correspondence is erratic. GEM2Net, on the other hand, is specific in its analysis, providing multiple distinct subcategories of biotic and abiotic stress conditions. This is contrary to ACT's global coexpression landscape analysis. Nevertheless, ACT's analysis using *AMS* as driver gene, produces results comparable to FlowerNet, proving that the analyses of ACT are not only solid at identifying ubiquitously expressed genes, but tissue specific genes as well. Furthermore, each subcategory of GEM2Net has a different sample pool which does not exceed two-digit numbers in a single case, limiting the consistency of the tool. Finally, its enrichment analysis results are complicated for the viewer and only provide specialized enrichment terms in many different stress categories instead of a single unanimous result table.

ACT identifies coexpressed genes to a user-selected gene of interest. It outputs a tree whose leaves consist of the driver gene and genes of similar expression patterns, implying participation in common biological processes and pathways. The properties of a gene of unknown function can be inferred by examining the subtree of coexpressed genes and their statistically significant overrepresented biological terms. When the driver gene has known partners and functions, ACT replicates known biology by “rediscovering” those genes and terms, a fact that validates ACT analysis. Different genes were used for the validation of ACT's gene coexpression analysis potential. For instance, since ribosome is a multimolecular complex, all ribosomal structural proteins are expected to be available during ribosome biogenesis. Thus, using a ribosomal protein gene as driver, a total of 134 genes that coded for structural constituents of ribosome were found clustered in the coexpression tree. Genes in chloroplast DNA are expected to be clustered in a correlation analysis as other tissues e.g., leaves, contain chloroplasts and others e.g., roots, do not. This hypothesis was verified by ACT since all available chloroplast genes were grouped together in a single clade. Examining genes related to cell wall biogenesis, revealed *VND7* as a key transcription factor which regulates the coexpressed genes, a finding that was already experimentally confirmed (Yamaguchi et al., 2011), while the *CTL2* gene, with a probable role in cell wall biosynthesis (Hossain et al., 2010; Koizumi et al., 2009) was grouped with proteins engaged in cellulose synthesis. Our results confirmed the coexpression of several diverse genes although functionally related by using driver specific genes such as *emb1692* (Daras et al., 2019).

In order to compare ACT with other related coexpression tools, such as ATTED-II, Genevestigator and Genemania, we established the same conditions by selecting the same number of top ranking genes coexpressed with *CTL2* for each tool and by using external tools such as, String and g:Profiler. ACT, ATTED-II, and Genevestigator exhibited similar String network densities, while Genemania produced the sparsest network (0.17). In g:Profiler analysis (which was chosen so that all enrichment analyses were based on the same gene reference list), ACT outperformed all its competitors, as its p value for the top enriched term which described *CTL2* was many orders of magnitude lower than that of the other tools. This suggests that ACT discovered a larger number of genes described by the “plant-type secondary cell wall biogenesis” term, proving that the gene hierarchical clustering approach performs better than the coexpression gene list creation and/or that the meticulous sample selection ultimately results in stronger gene correlations.

Concluding, the new version of ACT is not a mere incremental update over the previous version. Instead, it is essentially a new tool only inheriting the pivotal role of Pearson correlation coefficients. We anticipate that ACT can be a useful tool in the community of plant molecular biologists, as it serves as a starting point for creating experimentally verifiable hypotheses regarding functional partner discovery, gene function prediction, and regulatory role elucidation of transcription factors.

### Limitations of the study

The main limitation of ACT is inherited by the transcriptomic technology it is based on: Microarrays are unable to study the expression of genes for which no probe is available on the surface of the chip. In addition, cross-hybridization may distort the estimation of the correlation between members of the same family of genes and other genes, especially when default CDF is used. These issues are overcome by the use of RNA-seq which steadily replaces microarrays. Publicly available RNA-seq data for *A. thaliana* have exceeded that of microarray data, both in terms of quantity and quality. However, although RNA-seq has higher sensitivity, its output is highly comparable with that of microarrays, especially in average expression levels (Chen et al., 2017). Additionally, RNA-seq-based and microarray-based GCNs have been shown to produce similar correlation values (Malatras et al., 2020) and comparable biological pathway enrichments (Obayashi et al., 2018). RNA-seq has not yet replaced fully microarrays as the selection of the best normalization method for gene coexpression analysis is still up for debate. On the other hand, microarray normalization algorithms have been developed and perfected over the lifespan of this technology. Thus, the expression and coexpression of the genes is accurately estimated with microarrays. Furthermore, tools such as Expression Angler (Austin et al., 2016; Toufighi et al., 2005) and Arabidopsis eFP viewer (Winter et al., 2007), which are prominent in the plant biology community, are also fully based on microarrays. Another known limitation of ACT is its inability to portray anticoexpressed genes. Gene pairwise correlations are converted to non-negative distance value prior to hierarchical clustering. Thus, genes with anticorrelated expression profiles cannot be inferred. Furthermore, the coexpression tree depiction assumes that any gene may only be part of a single group of functional partners. This limitation of the hierarchical clustering methods contradicts known biology, where a gene may possess multiple “independent” functions. Finally, although there are hints to define the optimal tree size, its estimation may be to some degree subjective.

## STAR★Methods

### Key resources table

**Table:** 

REAGENT or RESOURCE	SOURCE	IDENTIFIER
**Deposited data**		

*Arabidopsis thaliana* microarray samples - ArrayExpress	(Kolesnikov et al., 2015)	https://www.ebi.ac.uk/arrayexpress/
*Arabidopsis thaliana* microarray samples - GEO	(Barrett et al., 2013)	https://www.ncbi.nlm.nih.gov/geo/
*Arabidopsis thaliana* microarray samples - NASCArrays	(Craigon et al., 2004)	http://bar.utoronto.ca/NASCArrays/index.php
Full list of microarray samples used for ACT	This paper	https://data.mendeley.com/datasets/hgvk669v89/

**Software and algorithms**		

Single channel array normalisation (SCAN)	(Piccolo et al., 2012)	https://www.bioconductor.org/packages/release/bioc/html/SCAN.UPC.html
Brainarray Custom CDF version 23	(Dai et al., 2005)	http://mbni.org/customcdf/23.0.0/ensg.download/pd.ath1121501.at.ensg_23.0.0.tar.gz
Simpleaffy	(Miller, 2018)	https://web.archive.org/web/20201024030658/http://www.bioconductor.org/packages/release/bioc/html/simpleaffy.html
affyQCReport	(Parman et al., 2021)	https://bioconductor.org/packages/release/bioc/html/affyQCReport.html
affyPLM	(Bolstad et al., 2005; Brettschneider et al., 2008)	https://bioconductor.org/packages/release/bioc/html/affyPLM.html
Phangorn version 2.5.5	(Schliep et al., 2017)	https://cran.r-project.org/web/packages/phangorn/index.html
WebGestalt	(Liao et al., 2019)	http://www.webgestalt.org/
String version 11	(Szklarczyk et al., 2021)	https://version-11-0.string-db.org/
Interact tree of life (iTOL) version 6	(Letunic and Bork, 2019)	https://itol.embl.de/
g:Profiler	(Raudvere et al., 2019)	https://biit.cs.ut.ee/gprofiler/gost
Flame	(Thanati et al., 2021)	http://flame.pavlopouloslab.info
Atted-II version 10	(Obayashi et al., 2018)	https://atted.jp/
Genevestigator	(Hruz et al., 2008)	https://genevestigator.com/
Genemania	(Franz et al., 2018)	https://genemania.org/
EXPath 2.0	(Chien et al., 2015)	http://expath.itps.ncku.edu.tw/index.html
PLANEX	(Yim et al., 2013)	http://planex.plantbioinformatics.org/
SeedNet	(Bassel et al., 2011)	http://netvis.ico2s.org/dev/seednet/#/
FlowerNet	(Pearce et al., 2015)	https://www.cpib.ac.uk/anther/
AtGGM2014	(Ma et al., 2015)	https://labs.plb.ucdavis.edu/dinesh-kumar/atggm2014.html
GEM2Net	(Zaag et al., 2015)	http://urgv.evry.inra.fr/GEM2NET/
Thalemine	(Krishnakumar et al., 2015)	https://bar.utoronto.ca/thalemine/begin.do
Gene ontology	(Gene Ontology Consortium, 2021)	http://geneontology.org/
Planteome	(Cooper et al., 2018)	https://planteome.org/
KEGG pathways	(Kanehisa and Goto, 2000)	https://www.genome.jp/kegg/pathway.html
AraCyc	(Schlapfer et al., 2017)	https://plantcyc.org/
WikiPathways	(Martens et al., 2021)	https://www.wikipathways.org/index.php/WikiPathways
AtRegNet	(Yilmaz et al., 2011)	https://agris-knowledgebase.org/moreNetwork.html
Plant Cistrome database	(O'Malley et al., 2016)	http://neomorph.salk.edu/dap_web/pages/index.php
Pfam	(Mistry et al., 2021)	http://pfam.xfam.org/
Cytoscape	(Shannon et al., 2003)	https://cytoscape.org/

### Resource availability

#### Lead contact

Further information and requests for resources should be directed to and will be fulfilled by the lead contact, Ioannis Michalopoulos (imichalop@bioacademy.gr).

#### Materials availability

This study did not generate new unique reagents.

### Method details

#### Expression data collection and processing

For coexpression analysis, all microarray data should be comparable to each other. Therefore, they need to originate from the same organism and the same type of chip and to be normalised with the same algorithm and the same parameters. ArrayExpress (Kolesnikov et al., 2015), GEO (Barrett et al., 2013) and NASCArrays (Craigon et al., 2004) public repositories were searched for *Arabidopsis thaliana* microarray experiments of all chip platforms. It was discovered that the most popular microarray chip in use is the Affymetrix Arabidopsis ATH1 Genome Array [GEO:GPL198; ArrayExpress:A-AFFY-2] representing more than 50% of the total microarray data volume. *Arabidopsis thaliana* ATH1 raw microarray data (CEL files) and their respective MIAME (Brazma et al., 2001) meta-data were programmatically downloaded from the aforementioned public repositories. After duplicate and corrupt sample removal, using an in-house PHP script, our dataset consisted of 19,887 unique microarray samples from 1390 studies. A suitable normalization algorithm was selected for this single channel microarray chip: The samples were normalized with the Single Channel Array Normalisation (SCAN) algorithm (Piccolo et al., 2012) using Brainarray Custom Chip Description File (version 23) (Dai et al., 2005). A MySQL relational database was designed to store all required data: gene expression values and metadata of each sample, as well as *Arabidopsis thaliana* gene description terms. Gene names and brief descriptions were downloaded from Thalemine (Krishnakumar et al., 2015), gene ontologies from Gene Ontology (Gene Ontology Consortium, 2021), plant ontologies from Planteome (Cooper et al., 2018), biological pathways from KEGG Pathways (Kanehisa and Goto, 2000), AraCyc (Schlapfer et al., 2017) and WikiPathways (Martens et al., 2021), transcription factor gene targets from AtRegNet (Yilmaz et al., 2011) and Plant Cistrome Database (O'Malley et al., 2016) and protein domains from Pfam (Mistry et al., 2021). Most of those data were programmatically downloaded, exploiting BioMart (Kinsella et al., 2011) XML-based data retrieval system, in the majority of the cases.

#### Quality control

Sample quality is decisive for a large-scale coexpression analysis. To eliminate low quality samples, a quality control strategy, similar to that of Muscle Gene Sets (Malatras et al., 2019), was conducted using simpleaffy (Miller, 2018), affyQCReport (Parman et al., 2021) and affyPLM (Bolstad et al., 2005; Brettschneider et al., 2008) packages of BioConductor suite (Gentleman et al., 2004; Huber et al., 2015) in R (R Core Team, 2019).For the Quality control step, primary data were normalised with MAS5.0 algorithm (Hubbell et al., 2002) using the Affymetrix default CDF. Affymetrix provides array quality metrics for each sample as well as general guidelines for the value thresholds, for example the percentage difference of present genes between samples of the same study should be no higher than 10% and 3′ to 5′ ratio of GAPDH and β-actin should not be higher than 1.25 and 3, respectively. As an additional quality control within series, Normalized Unscaled Standard Error (NUSE) and Relative Log Expression (RLE) multi-array metrics were used. NUSE boxplots should be centered at 1 with the low-quality samples centered above 1.1. RLE boxplots should be centered at near 0 and have similar spread with low-quality samples having an absolute spread higher than 0.2. Low-quality samples were identified primarily based on the output of RLE and NUSE. In the final step, whole plant or mutant samples were identified by examining the meta-data information of each sample and were manually removed. Eventually, 6933 distinct, wild-type, healthy samples were selected for coexpression analysis.

#### Gene coexpression tree creation

Pairwise sample correlations were calculated using Pearson Correlation Coefficient (*r*-values) (Pearson, 1895), using the expression values of 21,273 non-obsolete *Arabidopsis thaliana* genes, in the 6933 previously selected samples and a sample distance matrix was created using the *d = 1 – r* formula (Kassambara, 2017), resulting in a distance matrix, in Phylip format (Felsenstein, 2008), with a value range [0, 2] where the lowest value represents complete correlation and the highest value, complete anti-correlation. Based on the distance matrix, a sample correlation tree was created in Newick format (Archie et al., 2008), using Phangorn (Schliep et al., 2017) R package implementations of UPGMA (Sokal and Michener, 1958). On that tree, each leaf represented a unique sample. Since our main aim was the study of the global (i.e. tissue-independent) coexpression landscape of *Arabidopsis thaliana*, tissue bias had to be minimised by choosing the most representative samples of the entire dataset. Thus, the tree of 6933 sample-leaves, was programmatically pruned in an iterative procedure using an in-house algorithm trimming close leaves, where in each iteration the leaf with the shortest distance to its first common node was trimmed, leaving 3500 leaves which represent the most distinct samples. The gene expression values of the 3500 samples were used for the calculation of pairwise gene correlations as *r*-values and a gene distance matrix using the same *d = 1 – r* formula. Finally, the 21,273 gene-leaves coexpression tree was created, using UPGMA based on the distance matrix. That Newick-formatted tree constitutes the end product of the coexpression analysis and also the basis of ACT. To evaluate the resemblance between the distance matrix and the tree produced, the Cophenetic Correlation Coefficient (CPCC) (Farris, 1969), the correlation between the original distance matrix and the distance matrix represented by the tree (cophenetic matrix), was calculated. The cophenetic matrix was extracted using the *cophenetic* function from R stats package. The CPCC of our tree was 0.5923.

#### Web tool implementation

The web server is hosted on a Linux Ubuntu 18.04, 16-core, 64 GB memory system. A web-based user interface was created using HTML5 and CSS along with the Bootstrap library and certain JavaScript functions, such as the gene name and probe set ID auto-completion of the search field. All ACT scripts performing tasks such as the database connection, tree visualisation and enrichment analysis are written in PHP and run on an HTTPS protocol-verified Apache 2.4.29 web server.

An *Arabidopsis thaliana* gene is selected by the user, deemed the “driver” gene, and a gene coexpression subtree with 5 ancestral nodes is produced, based on the location of that driver gene on the gene coexpression tree. A scale bar, referring to *r*-values, is also displayed at the top of the subtree. The tree leaf names contain both the probe set ID and the official gene name. To define another probe set as the driver gene, the user clicks on this probe set ID, while clicking on a gene name redirects externally to the gene page entry in Thalemine. The tree size can also be altered producing a subtree with up to 25% of the total genes. The subtree can be downloaded in Newick format and can be viewed externally on the iTOL tree viewer (Letunic and Bork, 2019). Gene descriptions can be found on a table below.

By selecting any enrichment analysis from a drop-down menu, a relevant gene term over-representation analysis can be performed. The analysis is performed on the fly with the input being the genes depicted on the current subtree and over-represented biological terms (gene or plant ontologies, pathways, targeting transcription factors and protein domains) are displayed on the enrichment summary table. p value calculations are based on Hypergeometric Distribution (Forbes et al., 2011). Over-represented terms are ranked by their False Discovery Rate (FDR) (Benjamini and Hochberg, 1995) adjusted p values in ascending order. Only those terms with an FDR-adjusted p value ≤0.05 are presented. For each term, the hit percentage (times the term appearing in the coexpression subtree over its appearances in all available genes) and the over-representation rate (times observed over expected) are also presented. Increasing or decreasing the tree size affects the results of this analysis. By increasing the size of a tree, enriched terms that are not available in a smaller tree, may be revealed. On the other hand, a larger subtree may contain gene subclades of different functions, so decreasing the tree size would yield more specialised enriched term results. To this end, observing the fluctuations of biological term enrichment p values may also be helpful to determine the optimal tree size. In a second table, a full list of the genes of the subtree are displayed, along with all terms of that category that describe them, with links to their source website. Finally, the gene list of the subtree can also be downloaded to be used in subsequent analyses e.g. WebGestalt and links to STRING, Thalemine, g:Profiler and Flame websites are redirecting the gene list for additional analyses.

## Data Availability

• The microarray samples analyzed during the current study are available at:https://doi.org/10.17632/hgvk669v89.1 • ACT is freely available at www.michalopoulos.net/act • Any additional information required to reanalyse the data reported in this paper is available from the lead contact upon request.

## Supplemental information

Document S1. Figures S1–S5 and Tables S2–S4

Table S1. Descriptions of the correlated genes to *AT4G13170* which belongs to the L13 ribosomal family of proteins, related to Table 2
