# Supplementary material for: Arabidopsis Coexpression Tool: a tool for gene coexpression analysis in Arabidopsis thaliana
Source: iScience. 2021 Jul 10;24(8):102848. doi: 10.1016/j.isci.2021.102848 (PMC8334378; doi:10.1016/j.isci.2021.102848)
Supplement: Document S1. Figures S1–S5 and Tables S2–S4 [file mmc1.pdf]

iScience, Volume *24*

Supplemental information

Arabidopsis Coexpression Tool: a tool for gene coexpression analysis in *Arabidopsis thaliana*

Vasileios L. Zogopoulos, Georgia Saxami, Apostolos Malatras, Antonia Angelopoulou, Chih-Hung Jen, William J. Duddy, Gerasimos Daras, Polydefkis Hatzopoulos, David R. Westhead, and Ioannis Michalopoulos

Supplemental Figures 1-5

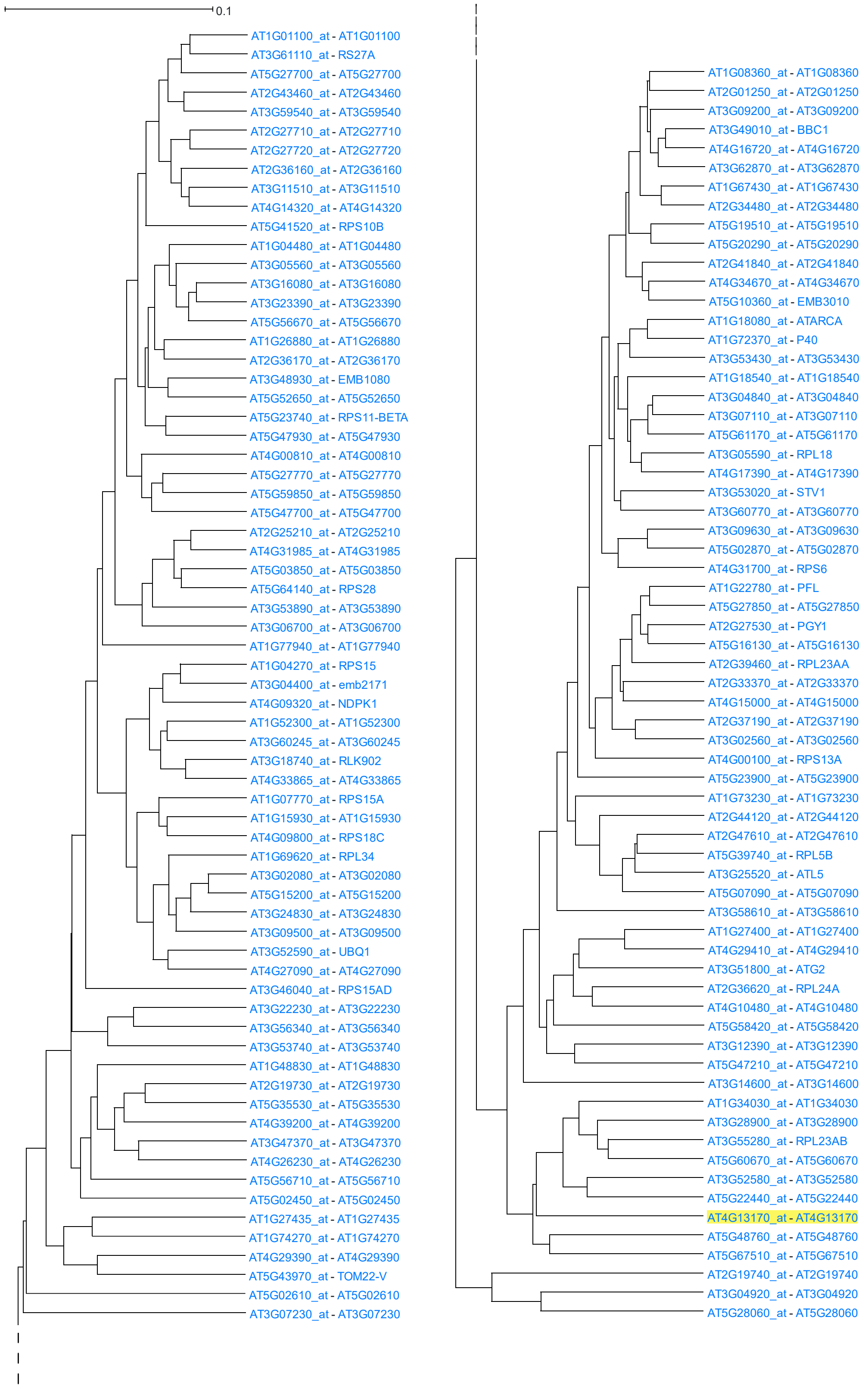

Figure S1. Coexpression subtree, as it is depicted in ACT website, produced using *AT4G13170* as a driver gene. The tree consists of 134 gene-leaves and the driver gene is highlighted in yellow background, related to Table 2.

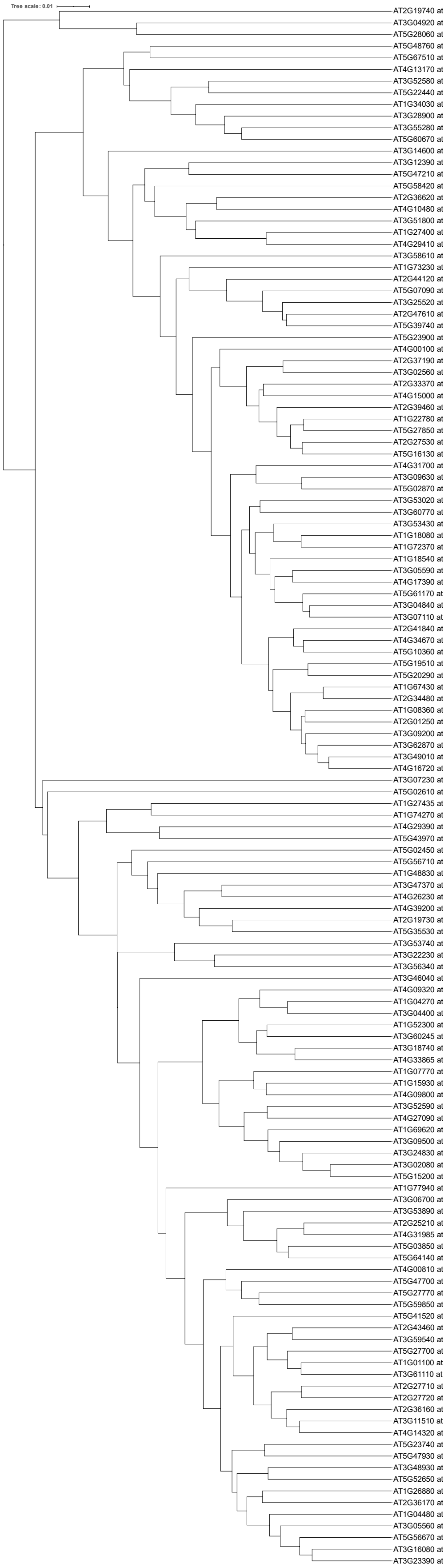

Figure S2. Coexpression subtree, as it is depicted in iTOL website, produced using *AT4G13170* as a driver gene in ACT, related to Table 2.

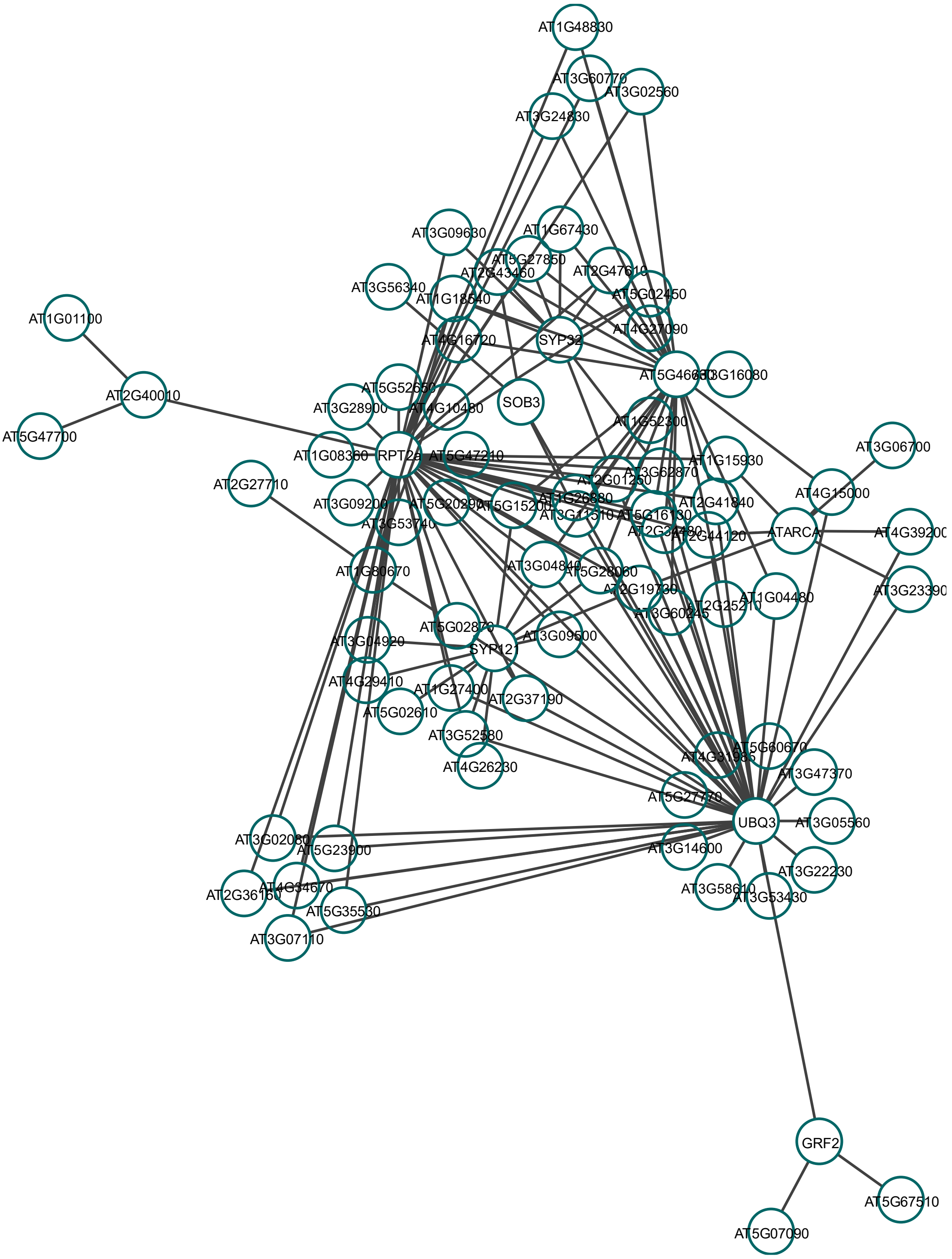

Figure S3. WebGestalt resulting gene network using coexpressed genes to *AT4G13170.*Network output of WebGestalt Network Topology-based analysis viewed by Cytoscape (Shannon et al., 2003), using the list of the coexpressed genes to *AT4G13170* from ACT's analysis as input. *UBQ3* is presented as the top-ranking neighbour, related to Table 2.

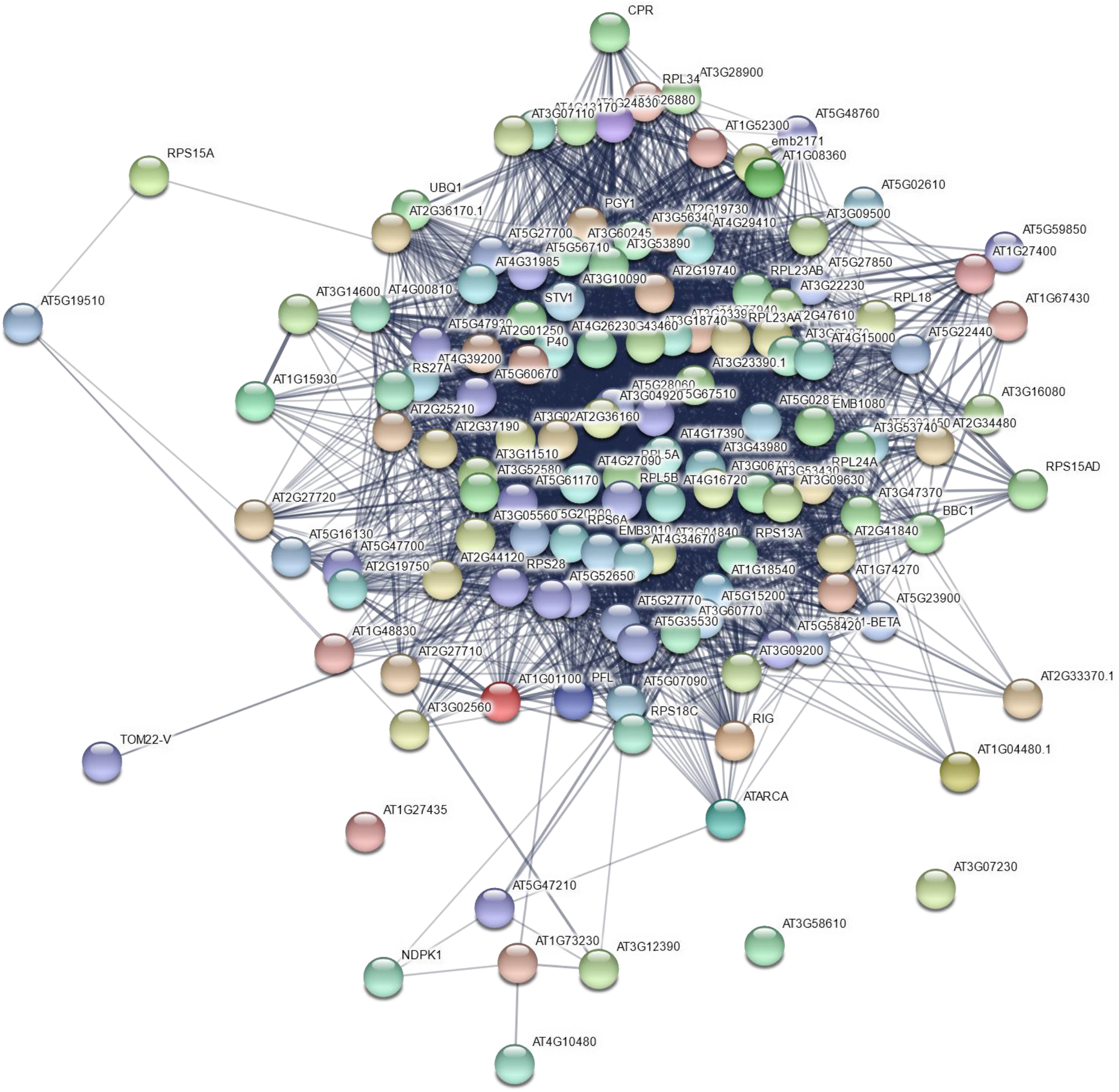

Figure S4. Protein-Protein association network output of STRING, using the list of the coexpressed genes to *AT4G13170* from ACT's analysis as input. The confidence lines are shown, only the text-mining option was selected along with medium confidence and no other interactions are included, related to Table 2.

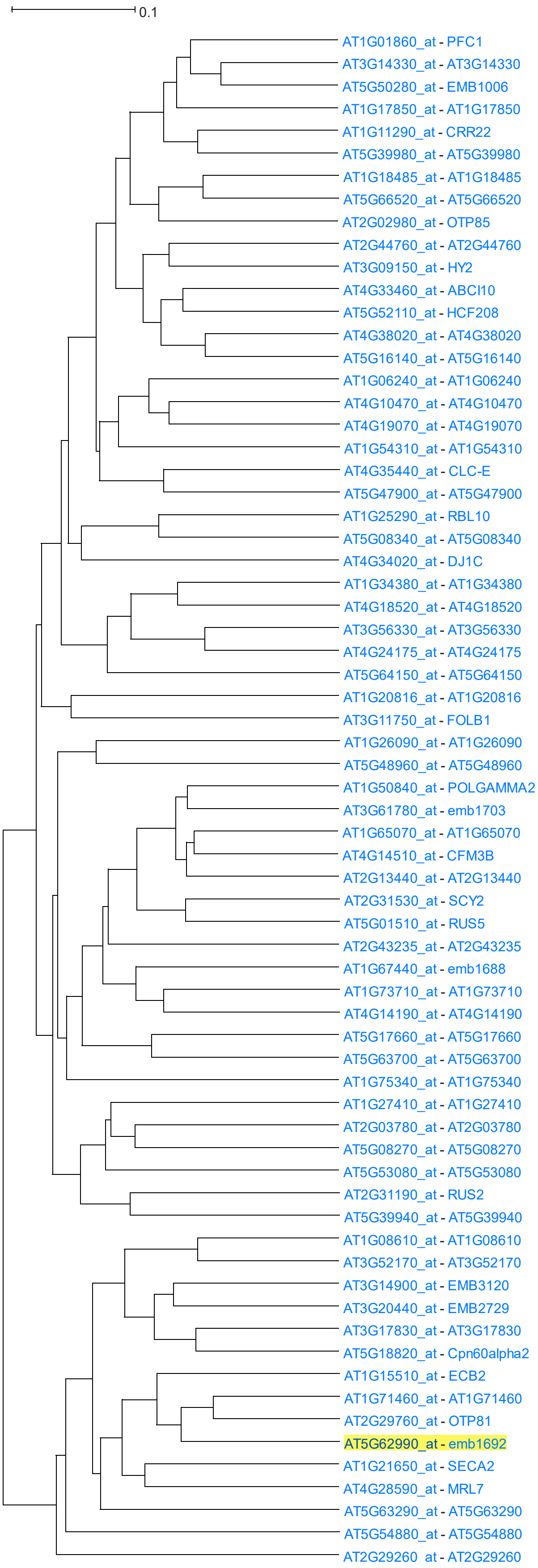

Figure S5. Resulting coexpression tree using *emb1692* as a driver gene after it was expanded to 8 ancestral nodes related to Table 7.

Supplemental Tables 2-4

Table S2. *HSP90* results of the over-representation analysis, related to Table 3.

Enrichment Summary for *HSP90*			
Category	p-value	Term ID	Description
GO: Biological Process	$9.1 \cdot 10^{-45}$	GO:0009409	response to heat
	$7.3 \cdot 10^{-34}$	GO:0009266	response to temperature stimulus
	$6.1 \cdot 10^{-19}$	GO:0009644	response to high light intensity
	$1.7 \cdot 10^{-18}$	GO:0042542	response to hydrogen peroxide
	$3.7 \cdot 10^{-18}$	GO:0009628	response to abiotic stimulus
	$8.5 \cdot 10^{-17}$	GO:0006457	protein folding
KEGG	$9.6 \cdot 10^{-25}$	ath04141	Protein processing in endoplasmic reticulum

Table S3. Enrichment analysis results for *COR15A*, related to Figure 1.

Enrichment Summary for *COR15A*			
Category	p-value	Term ID	Description
GO: Biological Process	$6.8 \cdot 10^{-6}$	GO:0009631	Cold acclimation

Table S4. Enrichment analysis results for *CEV1*, related to Figure 2.

Enrichment Summary for *CEV1*			
Category	p-value	Term ID	Description
GO: Biological Process	$6.4 \cdot 10^{-14}$	GO:0000271	polysaccharide biosynthetic process
	$1.4 \cdot 10^{-13}$	GO:0030244	cellulose biosynthetic process
	$1.9 \cdot 10^{-13}$	GO:0009833	plant-type primary cell wall biogenesis
	$1.9 \cdot 10^{-13}$	GO:0033692	cellular polysaccharide biosynthetic process
	$4.4 \cdot 10^{-13}$	GO:0051274	beta-glucan biosynthetic process
GO: Molecular Function	$5.8 \cdot 10^{-7}$	GO:0016760	cellulose synthase (UDP-forming) activity
	$1.7 \cdot 10^{-6}$	GO:0016759	cellulose synthase activity
	$2.6 \cdot 10^{-6}$	GO:0008172	S-methyltransferase activity
	$3.0 \cdot 10^{-6}$	GO:0008194	UDP-glycosyltransferase activity
GO: Cellular Component	$2.1 \cdot 10^{-19}$	GO:0005802	trans-Golgi network
	$1.1 \cdot 10^{-18}$	GO:0098791	Golgi subcompartment
